# nm23-H1 expression defines a high-risk subpopulation of patients with early-stage epithelial ovarian carcinoma

**DOI:** 10.1054/bjoc.2000.1116

**Published:** 2000-04-27

**Authors:** J Schneider, M Pollán, E Jiménez, K Marenbach, N Martínez, M Volm, D Marx, H Meden

**Affiliations:** Departamento de Especialidades Médico-Quirúrgicas, Universidad del País Vasco, Bilbao, Spain; Fundación Tejerina-Centro de Patología de la Mama, Madrid, Spain; Instituto Nacional de Epidemiología, Unidad de Epidemiología del Cáncer, Madrid, Spain; Clínica Universitaria de Navarra, Laboratorio de Oncología Molecular, Pamplona, Spain; Universitäts-Frauenklinik Göttingen, Göttingen, Germany; German Cancer Research Center, Heidelberg, Germany

**Keywords:** ovarian cancer, nm23, prognosis

## Abstract

The role of the nm23 gene in human ovarian cancer is still controversial. We studied the expression of the nm23-H1 gene in 247 human epithelial ovarian carcinomas. The patients were followed-up until their death, or for a minimum of 5 years if they survived. The expression of the gene was studied by means of immunohistochemistry and a semiquantitative scoring system considering the staining intensity and the number of reactive tumour cells. Patients carrying tumours with higher expression scores (4–6 on a scale from 0 to 6) had a significantly lower survival (*P* = 0.01) than the rest. Further stratified statistical analysis revealed that this effect was mainly attributable to the subgroup of patients with early-stage (I and II), well- and moderately differentiated tumours. In fact, a multivariate analysis carried out for this subset of patients showed nm23-overexpression to be the only significant independent predictor of an ominous prognosis. The association of nm23-overexpression with a worse prognosis was most probably not due to mutation of the nm23 gene, since mutational analysis in 60 tumours by means of single-strand conformational polymorphism and direct sequencing disclosed only one mutation, which was located outside the open reading frame. Our results seem to indicate that nm23 expression is associated with a significantly worse prognosis in early-stage, well-differentiated epithelial ovarian carcinoma, a finding with important clinical implications, considering that many patients with ovarian cancers showing these features do not undergo any further treatment beyond surgical staging. If confirmed, they could help in tailoring the treatment of these patients in the future. © 2000 Cancer Research Campaign


*British Journal of Cancer* (2000) **82**(10), 1662–1670
© 2000 Cancer Research Campaign
DOI: 10.1054/ bjoc.2000.1116, available online at http://www.idealibrary.com on IDEAL®

### nm23-H1 expression defines a high-risk subpopulation of patients with early-stage epithelial ovarian carcinoma

**J Schneider[1,2], M Pollán[3], E Jiménez[1,4], K Marenbach[1,5], N Martínez[1,6], M Volm[6], D Marx[5] and H Meden[5]**

[1]Universidad del País Vasco, Departamento de Especialidades Médico-Quirúrgicas, Bilbao, Spain; [2]Fundación Tejerina-Centro de Patología de la Mama, Madrid, Spain; [3]Instituto Nacional de Epidemiología, Unidad de Epidemiología del Cáncer, Madrid, Spain; [4]Clínica Universitaria de Navarra, Laboratorio de Oncología Molecular, Pamplona, Spain; [5]Universitäts-Frauenklinik Göttingen, Göttingen, Germany; [6]German Cancer Research Center, Heidelberg, Germany

**Summary** The role of the nm23 gene in human ovarian cancer is still controversial. We studied the expression of the nm23-H1 gene in 247 human epithelial ovarian carcinomas. The patients were followed-up until their death, or for a minimum of 5 years if they survived. The expression of the gene was studied by means of immunohistochemistry and a semiquantitative scoring system considering the staining intensity and the number of reactive tumour cells. Patients carrying tumours with higher expression scores (4–6 on a scale from 0 to 6) had a significantly lower survival ($P = 0.01$) than the rest. Further stratified statistical analysis revealed that this effect was mainly attributable to the subgroup of patients with early-stage (I and II), well- and moderately differentiated tumours. In fact, a multivariate analysis carried out for this subset of patients showed nm23-overexpression to be the only significant independent predictor of an ominous prognosis. The association of nm23-overexpression with a worse prognosis was most probably not due to mutation of the nm23 gene, since mutational analysis in 60 tumours by means of single-strand conformational polymorphism and direct sequencing disclosed only one mutation, which was located outside the open reading frame. Our results seem to indicate that nm23 expression is associated with a significantly worse prognosis in early-stage, well-differentiated epithelial ovarian carcinoma, a finding with important clinical implications, considering that many patients with ovarian cancers showing these features do not undergo any further treatment beyond surgical staging. If confirmed, they could help in tailoring the treatment of these patients in the future. © 2000 Cancer Research Campaign

**Keywords**: ovarian cancer; nm23; prognosis

The nm23 gene is a putative metastasis-suppressor gene originally identified by screening of cDNA libraries from murine melanoma cell sublines of varying metastatic potential (Steeg et al, 1988). Several evidences in favour of its metastasis suppressor capacity have been reported. A decrease in nm23-H1 RNA was observed in highly metastatic tumour cells from various rodent systems (Bevilacqua et al, 1989) and transfection of the nm23-H1 cDNA in murine melanoma and human breast cancer cells was shown to cause a reduction in cell migration in response to serum and defined growth factors in vitro and a reduction of tumour metastatic potental in vivo (Leone et al, 1991a).

The two highly homologous nm23 genes NME1 and NME2 are located in a tandem array 4 kb apart on the long arm of chromosome 17 (region 17q21.3) (Gilles et al, 1991), a region which is frequently deleted in cancer. NME1 and NME2 code for the proteins Nm23-H1 and Nm23-H2 respectively, of approximately 17 kDa each, which display an 88% amino acid homology between each other. It has been reported that these two proteins correspond to both chains of the nucleoside diphosphate kinase from human erythrocytes, (NDPK)A and (NDPK)B respectively (Gilles et al, 1991; Stahl et al, 1991).

*Received 16 September 1999*
*Revised 31 January 2000*
*Accepted 3 February 2000*

*Correspondence to:* J Schneider, Centro de Patología de la Mama, Calle José, Abascal 40, E-28003 Madrid, Spain nm23 seems to play an important role in human tumorigenesis, which has not been clearly defined up to now. Although Nm23 proteins have been demonstrated to have NDPK activity, both its precise function and the mechanism by which it supposedly participates in anti-metastatic protection in human tumours is still uncertain and controversial, since it has not been established whether the enzyme activity itself mediates the biological effect of nm23 in tumours or not. Potential roles for the Nm23 protein have been suggested, such as in the formation of a basement membrane (Howlett et al, 1994) tumour differentiation (Lombardi et al, 1995) and cell proliferation (Caligo et al, 1995).

Despite the high degree of homology between Nm23-H1 and Nm23-H2 proteins, they seem to have different functions. Nm23-H2 has been proposed as a co-factor, modulating the transcriptional activity of certain promoters, since it contains a putative leucine zipper motif in its sequence which shows high homology with purine binding factor (PuF), which has been shown to initiate transcription of the c-*myc* gene in vitro (Postel et al, 1993), with the I-factor, the ‘differentiation inhibiting factor’, purified from mouse myeloid leukaemia cells, which suppresses differentiation of leukaemic cells (Okabe-Kado et al, 1992) and with the neuroblastoma p19 protein (Hailat et al, 1991). Therefore, Nm23-H2 might be more involved in proliferation, and not in metastasis.

Loss of heterozygosity and/or reduced expression of nm23 has been correlated with worse prognosis and increased incidence of metastasis in many human tumours, such as breast carcinoma (Bevilacqua et al, 1989; Hennessy et al, 1991; Royds et al, 1993), malignant melanoma (Florenes et al, 1992), gastric cancer (Nakayama et al, 1993) and hepatocellular carcinoma (Nakayama et al, 1992), a finding that would agree with its putative role as a metastasis-suppressing or, more generally, as a tumour suppressor gene. On the other hand, however, we have that nm23-overexpression has been correlated with metastatic spread, progression and tumour aggressiveness in pancreatic carcinoma (Nakamori et al, 1993), lung adenocarcinoma (Ozeki et al, 1994), neuroblastoma (Hailat et al, 1991; Leone et al, 1993), colon carcinoma (Royds, 1994), and ovarian carcinoma (Mandai et al, 1994, 1995). A lack of correlation between NDP kinase expression and lymph node metastasis in breast carcinoma has also been suggested (Sastre-Garau et al, 1992). Moreover, in neuroblastoma, elevated levels of nm23 transcripts along with amplification and mutation of the nm23-H1 gene are associated with advanced stages of the disease and poor patient survival (Hailat et al, 1991; Leone et al, 1993; Chang et al, 1994). Therefore, it might be that nm23 plays a tissue-specific role and that different regulatory mechanisms may act depending on the kind of tumour.

Somatic allelic deletions of nm23-H1 have been studied in human cancers, such as breast, kidney, colon and lung (Leone et al, 1991b). Although in some of these cases somatic allelic deletions seem to correlate with an increased incidence of metastasis, it has not been possible to determine yet if this increase is actually related to the loss of nm23. Mutations and/or genetic alterations of the nm23-H1 gene, together with overexpression associated with a more malignant phenotype have also been found in some human tumours, like aggressive childhood neuroblastomas (Leone et al, 1993).

The role that the nm23-H1 gene might play in ovarian carcinoma has not been conclusively established up to now (Mandai et al, 1994, 1995; Scambia et al, 1996; Schneider et al, 1996). It has been reported by Mandai et al (1994) that ovarian cancer patients with metastatic lymph node involvement have lower nm23 levels than lymph node-negative cases, and by Kapitanovic et al (1995) that metastatic ovarian carcinomas were more frequently negative for nm23-H1 than non-metastatic ones. Mandai et al (1994) also studied the expression of both nm23-H1 and nm23-H2 in ovarian tumours and found significantly higher levels of mRNA in carcinoma tissues, compared with benign tumours. This was confirmed recently by us, who studied nm23 expression in ovarian cancer by means of immunohistochemistry in a small, pilot study, and found that most invasive, advanced-stage tumours overexpressed nm23-H1, whereas low-malignant-potential stage I ovarian tumours did so significantly less (Schneider et al, 1996).

We devised the present study in order to determine whether the changes in nm23 expression influence prognosis and are associated with genetic alterations.

### MATERIALS AND METHODS

### Patients and tumours

We studied 247 epithelial ovarian carcinoma samples from Universitäts-Frauenlink Göttingen, Germany, Universidad del País Vasco, Bilbao, Spain, and German Cancer Research Center, Heidelberg, Germany, belonging to patients treated between 1982 and 1994.

All patients were submitted initially to a complete staging laparotomy. Lymphadenectomy was not routinely performed in advanced stages, since it would not alter the final staging (tumour masses above 2 cm in diameter define the same IIIC stage as positive retroperitoneal nodes), and thus would not influence the kind of adjuvant treatment. It was limited to apparent early stages, to confirm that they were not occult surgical stage IIIC. The archival pathologic specimens of this initial laparotomy yielded the tumour material for the present study.

The histology of the tumours was as follows: 102 (41.3%) serous-papillary; 47 (19.0%) endometrioid; 37 (15.0%) mucinous; 48 (19.4%) undifferentiated; 12 (4.9%) clear cell; 1 (0.4%) mixed-epithelial.

Staging of the patients was performed according to the classification of the International Federation of Obstetrics and Gynaecology (FIGO). The distribution by surgical stage was: 28 (11.3%) stage I; 29 (11.7%) stage II; 144 (58.4%) stage III; 46 (18.6%) stage IV.

From surgical stage IG3 upwards, all patients received platinum-based adjuvant chemotherapy, which was maintained for a minimum of 6 cycles if their status allowed it, or if there was no clinically evident tumour progression after the first three cycles of treatment.

Immunohistochemistry was performed on 247 ovarian tumours. Sixty samples were also studied for mutations in the nm23-H1 gene by means of polymerase chain reaction single-strand conformational polymorphism (PCR-SSCP) analysis and direct sequencing.

### Immunohistochemistry

Expression of the nm23-H1 gene was analysed by means of immunohistochemistry in all 247 tumour samples, looking for the typical cytoplasmic reaction described in previous cellular localization studies.

The immunohistochemical technique was the same described extensively by us in a previous pilot study on nm23 expression in ovarian tumours (Schneider et al, 1996). Briefly, the procedure was carried out on 5 μm sections from routinely processed, formalin-fixed, paraffin-embedded tumour blocks. The technique itself was a variant of the usual streptavidin–biotin–peroxidase method previously described by us for fresh-frozen tissue (Schneider et al, 1994*a*). To ensure uniformity of results we used a commercial streptavidin–biotin–peroxidase kit (Histostain-SP, Zymed, San Francisco. CA, USA) throughout the whole procedure, which was entirely carried out at room temperature. As positive controls we used slides from mammary carcinomas known to express high levels of nm23. The monoclonal antibody used was NCL-nm23, manufactured by Novocastra Ltd (Newcastle, UK). NCL-nm23 recognizes the nm23-H1 protein, with a reported slight cross-reactivity for nm23-H2.

The slides were dewaxed in xylene, and then rehydrated in descending concentrations of alcohol (100%, 96% and then 70%) and finally 10 min in phosphate-buffered saline (PBS). Afterwards, the preparations were incubated with blocking serum for 10 min, and subsequently with the monoclonal antibody NCL-nm23 diluted 1:100, for 1 hour in a humid chamber. They were then washed in PBS three times for 3 min, after which the second biotinylated-bridge-antibody was applied for 10 min. Following three washes in PBS, 3 min each, the slides were incubated with the streptavidin–peroxidase complex for 10 min, washed again three times in PBS and stained with either diaminobenzidine or aminoethyl carbazole for 3 min. They were then counterstained with haematoxylin for 30 s, and mounted. Slides from each tumour

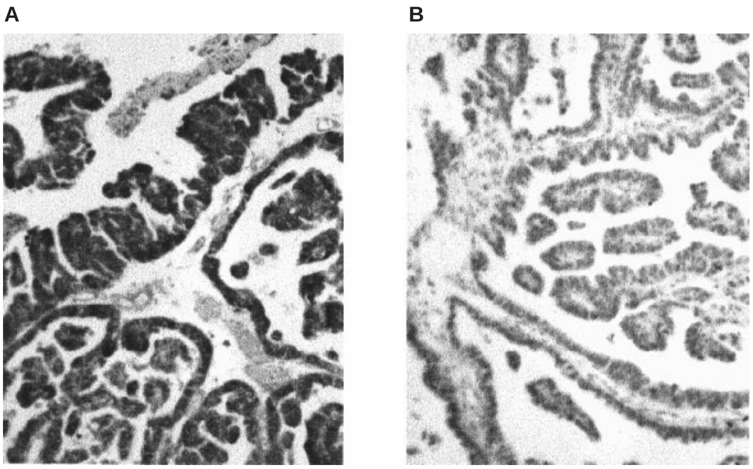

**Figure 1** (**A**) Immunohistochemical detection of nm23-H1 expression. Strong staining in more than 50% of tumour cells. NCL-nm23 MAb, streptavidin–biotin–peroxidase × 250. (**B**) Negative control of (**A**)

**Table 1** Primers used for amplification of exons 1 through 5 of the nm23 gene and amplification conditions employed

| Exon | Size (bp) | Annealing t (°C) | Sequence |
|---|---|---|---|
| 1 | 79 | 55 | PU:5′-GTCTGAAAAACGTAGCGCCGG-3′<br>PD:5′-CTTAGGTTTGAACTCCGGCTG-3′ |
| 2 | 130 | 58 | PU:5′-GCTTGAGACGGATGACGCTGTA-3′<br>PD:5′-CAGGTTAATCACAGTGTTCTCC-3′ |
| 3 | 101 | 58 | PU:5′-ATGTCCTTAGATGGTTTGGGGGT-3′<br>PD:5′-TTTGGTCTCATTCATGGCTGTAT-3′ |
| 4 | 113 | 50 | PU:5′-GCCACATTTTCTGCTGTGATT-3′<br>PD:5′-CCCAAATCCTTGTGGCAACT-3′ |
| 5 | 115 | 55 | PU:5′-GTCTAATGTCCATGGAGCTTC-3′<br>PD:5′-CAGATGGTCGGGGATGGTAAC-3′ |

were processed in parallel in identical fashion, but omitting the monoclonal antibody and leaving them with the blocking serum instead, and served as negative controls.

For the evaluation of the results of this study, we have adopted the semiquantitative scale used previously by us (Schneider et al, 1994*a*, 1996) which takes into account both the strength of the staining reaction, as well as the proportion of reactive tumour cells. So, + stands for staining of lower intensity than the positive control, and ++ for staining of equal or higher intensity than the positive control. Isolated tumour cells or tumour cell groups, comprising less than 10% of visible tumour cells were termed ‘a’, with ‘b’ designating up to 50% reactive tumour cells, and ‘c’ very numerous (50–100%) reactive cells. Finally, a numerical value was attributed to each possible combination: ‘+a’ 1 point, ‘++a’ 2 points, ‘+b’ 3 points, ‘++b’ 4 points, ‘+c’ 5 points and finally ‘++c’ 6 points. In this way, statistical processing of the data is made more easy and, taking into account the staining intensity and the number of reactive cells, the figure obtained reflects in an acceptable way the overall level of expression of the studied gene in each particular tumour. An example of ‘++c’ staining (highest score of 6 points) is given in Figure 1.

### Analysis of mutations by PCR-SSCP

The open reading frame of nm23-H1 is composed of five exons, displayed along a genomic DNA fragment of approximately 10 kb. They were amplified by means of PCR and mutational analysis of the gene was performed by means of SSCP in 60 ovarian tumours.

We employed a simplified, non-radioactive variant of the PCR-SSCP technique previously described by us for determining p53 mutations in gynaecologic tumours (Schneider et al, 1994*b*). It is, in its turn, a modification of a previously reported protocol (Martínez et al, 1997) and is performed on 7.5 × 10 × 0.5 cm non-denaturing polyacrylamide gels.

DNA was extracted both from fresh-frozen, or from paraffin-embedded tissue samples as previously described (Schneider et al, 1994*b*). For the PCR amplification reaction we used the oligonucleotide primers flanking each exon described by Bafico et al (1993) with the annealing temperature being adjusted for each primer pair (Table 1). The PCR reaction mix (25 μl) contained approximately 100 ng DNA, 50 pmoles of each primer, 1.25 U Taq polymerase, 100 mM of each deoxyribonucleoside triphosphate, 1.5 mM magnesium-chloride ($MgCl_2$) 1 × reaction buffer and $ddH_2O$ up to 25 μl. The three-step PCR was carried out for 30 cycles consisting of a denaturing step at 95°C for 1 min, followed by 30 s at the optimal annealing temperature for each primer pair, and 72°C for 30 s as final extension step. The obtained amplified product was then denatured as follows:

1–1.5 μl of PCR-product were added to 7 μl of the denaturalizing solution which contains 95% formamide, 20 mM EDTA, 0.05% bromophenol blue and 0.05% xylene cyanol. Afterwards the mixture was heated to 95°C for 10 min, and then rapidly

*© 2000 Cancer Research Campaign*

**Table 2** Predictors of survival in ovarian cancer according to the univariate analysis

| | No of patients | No of deaths | % of Survival 12 months | 36 months | 60 months | Hazard ratio | 95% | Confidence interval | *P*-value |
|---|---|---|---|---|---|---|---|---|---|
| Age | | | | | | | | | |
| < 60 | 102 | 74 | 79 | 55 | 36 | 1 | | | |
| 60–70 | 66 | 53 | 70 | 35 | 27 | 1.43 | 1.00 | 2.03 | 0.048 |
| ≥ 70 | 43 | 37 | 60 | 26 | 18 | 1.88 | 1.27 | 2.76 | 0.002 |
| Unknown | 36 | 25 | 78 | 38 | 22 | 1.48 | 0.93 | 2.34 | 0.096 |
| Histologic type | | | | | | | | | |
| Serous | 102 | 81 | 72 | 37 | 23 | 1 | | | |
| Mucinous | 37 | 23 | 73 | 57 | 42 | 0.58 | 0.36 | 0.92 | 0.021 |
| Mixed-epithelial | 1 | 1 | 100 | 100 | 0 | — | | | |
| Undifferentiated | 48 | 38 | 75 | 39 | 24 | 0.93 | 0.64 | 1.37 | 0.730 |
| Endometrioid | 47 | 35 | 79 | 53 | 41 | 0.70 | 0.47 | 1.04 | 0.076 |
| Clear cell | 12 | 11 | 58 | 21 | 10 | 2.09 | 1.11 | 3.94 | 0.023 |
| Surgical stage | | | | | | | | | |
| I | 28 | 9 | 89 | 81 | 73 | 1 | | | |
| II | 29 | 15 | 93 | 72 | 58 | 1.69 | 0.74 | 3.86 | 0.215 |
| III | 144 | 122 | 71 | 36 | 20 | 5.17 | 2.59 | 10.31 | <0.001 |
| IV | 46 | 43 | 59 | 20 | 11 | 7.67 | 3.68 | 15.99 | <0.001 |
| Grade of differentiation | | | | | | | | | |
| Well | 22 | 13 | 68 | 59 | 50 | 1 | | | |
| Moderate | 47 | 29 | 89 | 61 | 52 | 1.00 | 0.52 | 1.93 | 0.995 |
| Poor | 147 | 125 | 69 | 34 | 19 | 2.08 | 1.17 | 3.70 | 0.013 |
| Unknown | 31 | 22 | 74 | 37 | 22 | 2.12 | 1.06 | 4.26 | 0.034 |
| Index of Nm23-H1 expression | | | | | | | | | |
| 0–3 | 135 | 94 | 73 | 46 | 36 | 1 | | | |
| 4–6 | 112 | 95 | 73 | 38 | 20 | 1.46 | 1.10 | 1.94 | 0.010 |

plunged into ice for at least 5 min, prior to loading it onto the electrophoresis gel, which was a non-denaturing 15% polyacrylamide minigel (7.5 × 10 × 0.5 cm) (acrylamide:bisacrylamide = 49:1). The buffer chamber was filled with 0.5 × TBE buffer.

The wells were loaded with approximately 8 μl of the sample mixture described above, and the gel was run in a Mini Protean (BioRad) vertical electrophoretic chamber at 300–400 V under 4°C refrigeration for 2–3 h, according to the specifications displayed in Table 2, which varied for each exon, in order to optimize the visualization of the bands. Finally, the gels were silver-stained at room temperature: (i) 3 min incubation in 1% nitric acid ($HNO_3$); (ii) brief washing in distilled water; (iii) 25 min incubation, with gentle agitation, in 0.1% silver nitrate solution ($AgNO_3$) with 150 μl formaldehyde 37% per 100 ml solution; (iv) brief washing in distilled water; (v) incubation in cold 3% $NaCO_3$ solution with 150 μl formaldehyde 37% per 100 ml solution; the solution is discarded as soon as it blackens, and fresh solution is added to repeat the procedure at least once again or until the bands appear clearly in the gel; (vi) when the staining is considered optimal, the reaction is stopped by incubating the gel in 10% acetic acid for 30 min; (vii) the gels can then be dessicated for permanent storage.

Bands should be clearly visible, with low or absent background. Mutations make themselves evident by a shift in the migration pattern of the bands compared to the normal counterparts, an aberrant number of bands, or a combination of both. Direct sequencing of the amplified exons was performed using the ABI Prism 377 DNA Sequencer by Perkin-Elmer, strictly following the manufacturer's protocol, using their reagents.

### Statistical analysis

Time was calculated from the date of surgery to the date of either death or last follow-up visit prior to closure of the study. It was ensured that surviving patients were indeed alive at this time. If this could not be done, the patients were included as 'lost' following their last visit. The median follow-up time for the whole cohort (dead and surviving) was 27 months (range 1–171).

The association between survival and nm23 overexpression was investigated using the Kaplan–Meier method and the log-rank test. The prognostic importance of this variable was assessed in a Cox proportional hazards regression analysis in the whole cohort, and also separately in each stratum corresponding to the other predictive variables identified in the univariate analysis. Finally, a multivariate model was fitted, including nm23 status and the rest of variables proving to have an independent prognostic effect. In the final model, we tested whether any interaction term involving nm23 expression was statistically significant.

Implicit in Cox's model is the assumption of proportional hazards, namely, a stable prognostic effect over time. Given the extensive follow-up of this study, we considered it important to check if this condition was indeed true for the studied marker. For this purpose, crude and adjusted analyses were obtained at different time intervals, as proposed by Breslow and Day (1987). In addition, a graphical evaluation was carried out, by plotting the cumulative hazard ratio along the follow-up period (Hess, 1995). This parameter is a reasonable estimate of the hazard ratio and, due to its greater stability, easier to plot on a graph. The graph gives an intuitive image of the stability of the marker's predictive power over time.

© 2000 Cancer Research Campaign 

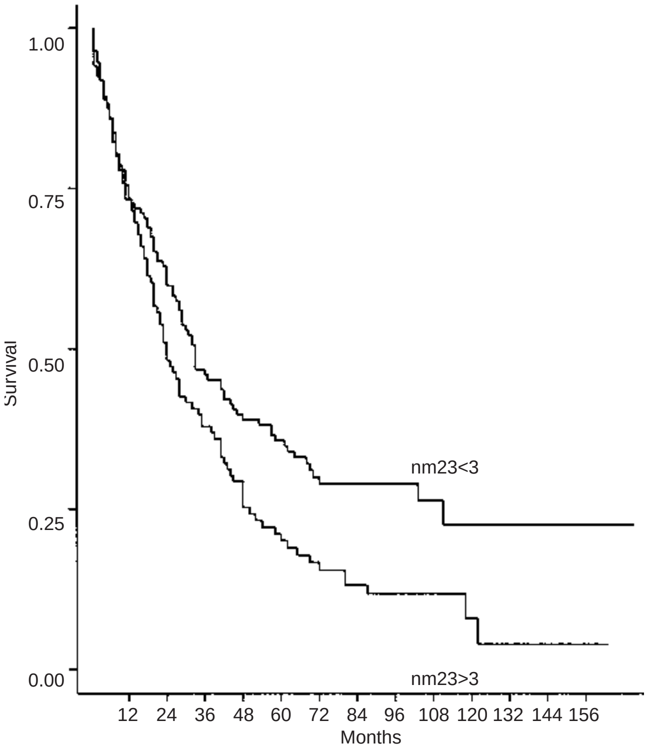

**Figure 2** Life table analysis according to nm23 expression in epithelial ovarian cancer. *n* = 247

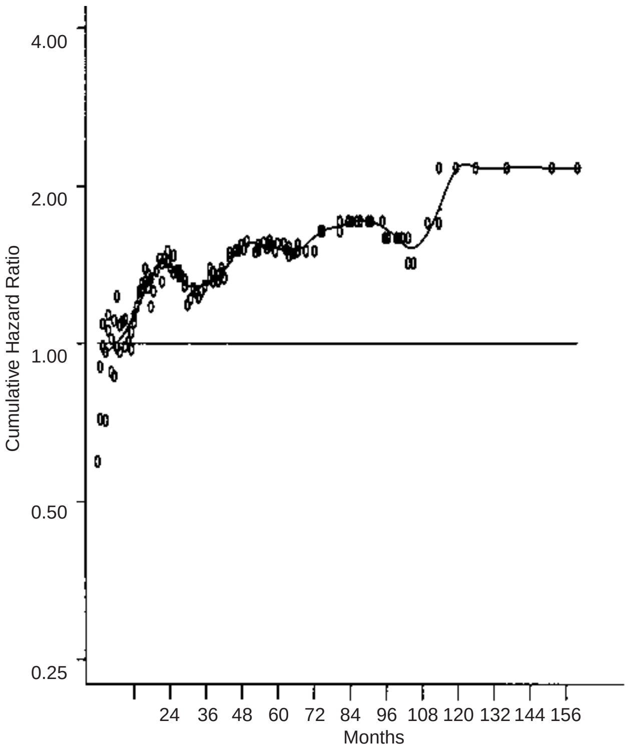

**Figure 3** Graphical representation of the cumulative hazard ratio of nm23 expression in epithelial ovarian cancer along the follow-up period. A constant positive value represents a stable negative prognostic effect over time

As a last step, a multivariate model was used to evaluate a more restricted and homogeneous set of cases, including only well or moderately differentiated tumours corresponding to surgical stages I and II.

### RESULTS

Ovarian carcinoma samples from 247 patients were examined, prior to any kind of chemotherapeutic or hormonal treatment, for the expression of the nm23-H1 gene.

Mutations were studied by means of PCR-SSCP and direct sequencing of the open reading frame of the gene, in a subset of 60 randomly chosen tumours.

In the univariate analysis (Table 2), the most discriminating cut-off level was obtained for the group of tumours showing a strong staining in more than 10% of cells (++b, +c or ++c, or with scores 4–6, according to our semiquantitative scale). When comparing this group of patients to the rest, they showed a significantly worse survival (Figure 2). The effect of nm23 overexpression on prognosis, moreover, was maintained over the whole follow-up period (Figure 3). The stratified univariate analysis, looking for prognostically relevant associations between nm23 overexpression and other predictive parameters (Table 3), seemed to indicate a strong discriminating power of nm23 in the subgroup of well- and moderately differentiated early (I & II) stage tumours. This was confirmed by stratified life-table analysis (Figure 4), as well as by multivariate analysis, which demonstrated that, in this subset of patients with an inherent ‘better’ prognosis, the only independent predictor of an ominous outcome was, indeed, nm23 overexpression (Table 5). In the complete multivariate model, contemplating the whole cohort of patients, on the other hand (Table 4), the predictive power of nm23 was overshadowed by the classically known independent prognosticators: age > 60 years, advanced surgical stage and poor degree of differentiation.

Mutation of the nm23 gene, as suggested by an abnormal band pattern in the PCR-SSCP analysis, was detected in only one tumour out of 60 analysed by this means. This mutation (a C to A transversion) was located in the untranslated region within exon 1 of the nm23-H1 gene, 78 base pairs before the starting codon. Therefore, it can be assumed that the negative impact on prognosis of nm23 overexpression is most probably due to the effect of the wild-type gene in our series of ovarian cancers, and not to mutated variants of it.

### DISCUSSION

The real function of the nm23-H1 gene in most human tumours is still uncertain, and ovarian carcinoma is no exception in this respect.

According to Mandai et al (1994), who were the first to study nm23 expression in ovarian cancer by means of mRNA measurements, overexpression of both nm23-H1 and nm23-H2 genes seems to be associated with tumour progression, since there are higher levels of expression in malignant tissue with respect to

 *© 2000 Cancer Research Campaign*

**Table 3** Predictors of survival in ovarian cancer according to nm23-H1 expression; stratified analysis.

| | No of patients | No of deaths | % Survival 12 months | % Survival 36 months | % Survival 60 months | Hazard ratio | 95% Confidence interval | *P*-value |
|---|---|---|---|---|---|---|---|---|
| Age | | | | | | | | 0.347 |
| < 60 | | | | | | | | |
| nm23: 0–3 | 61 | 40 | 75 | 52 | 41 | 1 | | |
| nm23: 4–6 | 41 | 34 | 85 | 59 | 29 | 1.29 | 0.81–2.03 | 0.282 |
| 60–70 | | | | | | | | |
| nm23: 0–3 | 43 | 33 | 74 | 37 | 29 | 1 | | |
| nm23: 4–6 | 23 | 20 | 61 | 30 | 21 | 1.29 | 0.74–2.25 | 0.375 |
| ≥ 70 | | | | | | | | |
| nm23: 0–3 | 22 | 17 | 59 | 41 | 31 | 1 | | |
| nm23: 4–6 | 21 | 20 | 62 | 10 | 5 | 1.89 | 0.96–3.74 | 0.067 |
| Unknown | | | | | | | | |
| nm23: 0–3 | 9 | 4 | 89 | 57 | 38 | 1 | | |
| nm23: 4–6 | 27 | 21 | 74 | 33 | 18 | 2.32 | 0.79–6.84 | 0.126 |
| Histologic type | | | | | | | | <0.001 |
| Serous | | | | | | | | |
| nm23: 0–3 | 55 | 39 | 69 | 40 | 32 | 1 | | |
| nm23: 4–6 | 47 | 42 | 74 | 33 | 12 | 1.42 | 0.92–2.20 | 0.115 |
| Mucinous | | | | | | | | |
| nm23: 0–3 | 33 | 19 | 79 | 60 | 47 | 1 | | |
| nm23: 4–6 | 4 | 4 | 25 | 25 | – | 4.60 | 1.50–14.11 | 0.008 |
| Undifferentiated | | | | | | | | |
| nm23: 0–3 | 20 | 16 | 70 | 40 | 25 | 1 | | |
| nm23: 4–6 | 28 | 22 | 79 | 38 | 23 | 1.13 | 0.59–2.15 | 0.716 |
| Endometrioid | | | | | | | | |
| nm23: 0–3 | 23 | 16 | 83 | 48 | 39 | 1 | | |
| nm23: 4–6 | 24 | 19 | 75 | 58 | 43 | 1.12 | 0.58–2.19 | 0.730 |
| Clear cell | | | | | | | | |
| nm23: 0–3 | 4 | 4 | 50 | 25 | 25 | 1 | | |
| nm23: 4–6 | 8 | 7 | 62 | – | – | 1.07 | 0.27–4.21 | 0.921 |
| Surgical stage | | | | | | | | 0.010 |
| I | | | | | | | | |
| nm23: 0–3 | 22 | 7 | 95 | 86 | 75 | 1 | | |
| nm23: 4–6 | 6 | 2 | 67 | 67 | 67 | 1.21 | 0.25–5.95 | 0.811 |
| II | | | | | | | | |
| nm23: 0–3 | 20 | 8 | 90 | 70 | 70 | 1 | | |
| nm23: 4–6 | 9 | 7 | 100 | 77 | 27 | 2.17 | 0.78–6.04 | 0.139 |
| III | | | | | | | | |
| nm23: 0–3 | 70 | 57 | 69 | 33 | 21 | 1 | | |
| nm23: 4–6 | 74 | 65 | 73 | 39 | 19 | 1.04 | 0.73–1.49 | 0.812 |
| IV | | | | | | | | |
| nm23: 0–3 | 23 | 22 | 52 | 26 | 13 | 1 | | |
| nm23: 4–6 | 23 | 21 | 65 | 13 | 9 | 1.13 | 0.61–2.06 | 0.701 |
| Differentiation | | | | | | | | 0.001 |
| Well or moderate | | | | | | | | |
| nm23: 0–3 | 52 | 28 | 85 | 65 | 59 | 1 | | |
| nm23: 4–6 | 17 | 14 | 76 | 47 | 27 | 2.06 | 1.08–3.94 | 0.028 |
| Poor | | | | | | | | |
| nm23: 0–3 | 75 | 62 | 64 | 62 | 20 | 1 | | |
| nm23: 4–6 | 72 | 63 | 74 | 67 | 18 | 0.99 | 0.70–1.42 | 0.972 |
| Unknown | | | | | | | | |
| nm23: 0–3 | 8 | 4 | 88 | 49 | 0 | 1 | | |
| nm23: 4–6 | 23 | 18 | 70 | 33 | 22 | 1.56 | 0.52–4.69 | 0.431 |

normal ovary and benign tumours. This has been subsequently confirmed by other studies (Leary et al, 1995; Schneider et al, 1996) with similar results using either Northern blotting or immunohistochemistry. Although Mandai et al (1994) together with Viel et al (1995) report that nm23 overexpression shows a significant association with more advanced tumour stages, according to them it also seems to exert a suppressive action on the development of lymph node metastasis. This apparently contradictory finding is still devoid of a plausible explanation. Our study, unfortunately, does not throw any additional light on this particular point. As has been explained in Materials and Methods, we did not routinely perform lymphadenectomies in advanced stages, for this would have only increased the operative time and the surgical risk, without influencing therapeutic decisions, according to our treatment protocols.

Scambia et al (1996) showed an association of nm23-H1 overexpression with both survival advantage and greater response to chemotherapy in patients with advanced ovarian cancer, the percentage of nm23-H1 positivity being higher in lymph node-negative (70%) than in lymph node-positive cases (40%). They studied 106 samples by immunohistochemistry and they found that the survival rate of nm23-H1-positive cases was 50% versus

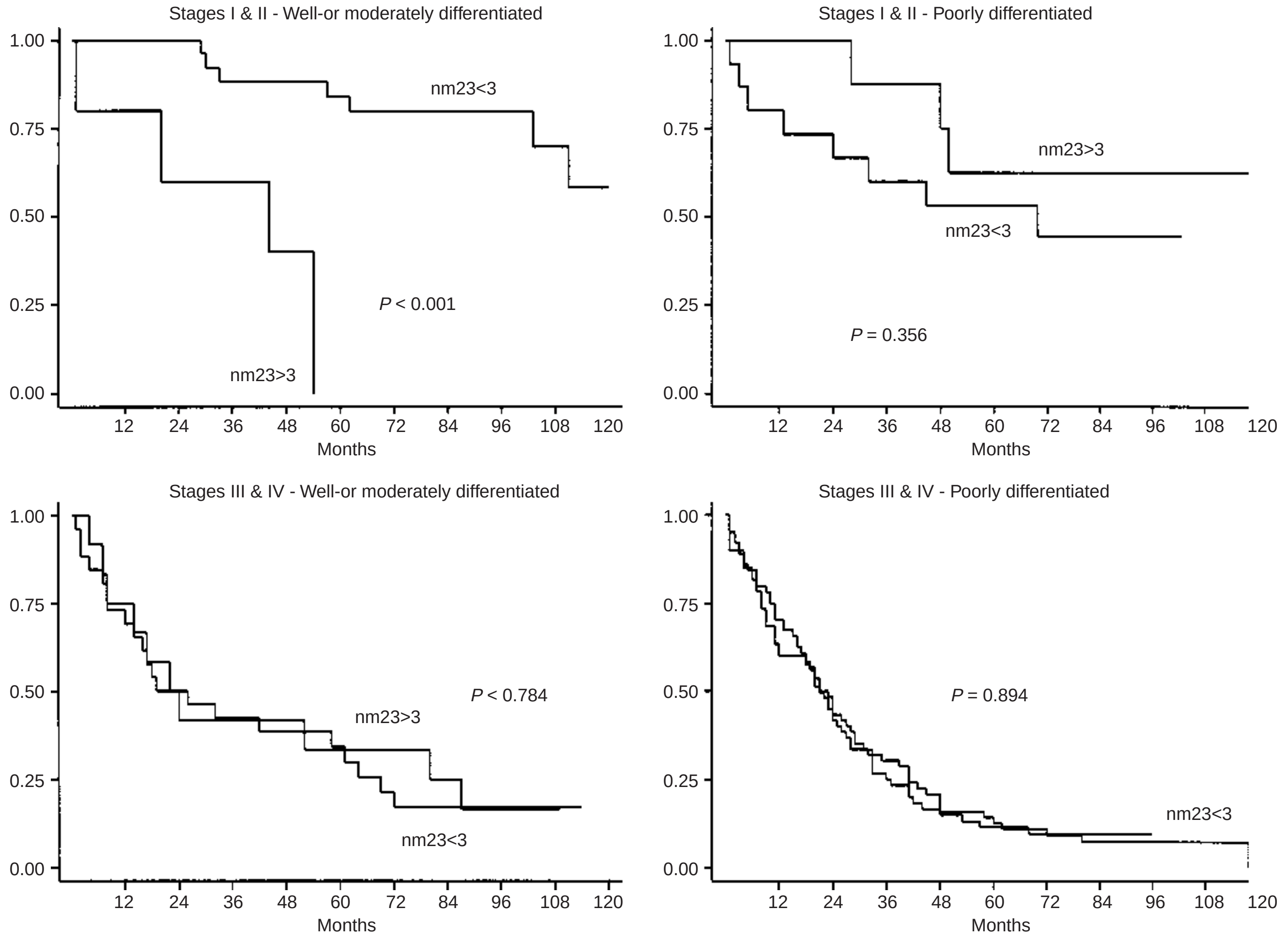

**Figure 4** Life table analysis of the effect of nm23 expression in different subsets of patients, stratified by stage and differentiation of the tumours

**Table 4** Predictors of survival in ovarian cancer according to the multivariate analysis

| | Results considering the whole cohort | | | Results considering only women with known age | | |
|---|---|---|---|---|---|---|
| Variable | Hazard ratio | 95% Confidence interval | *P*-value | Hazard ratio | 95% Confidence interval | *P*-value |
| Differentiation & nm23 expression | | | | | | |
| Well-moderate & nm23=0–3 | 1 | | | 1 | | |
| Well-moderate & nm23=4–6 | 1.43 | 0.76–2.69 | 0.269 | 1.61 | 0.83–3.11 | 0.160 |
| Poor | 1.53 | 1.05–2.23 | 0.025 | 1.77 | 1.15–2.72 | 0.009 |
| Surgical stage | | | | | | |
| I | 1 | | | 1 | | |
| II | 1.46 | 0.66–3.54 | 0.316 | 1.65 | 0.69–3.93 | 0.259 |
| III | 4.56 | 2.25–9.27 | <0.001 | 4.75 | 2.24–10.1 | <0.001 |
| IV | 5.88 | 2.76–12.5 | <0.001 | 6.71 | 2.71–13.6 | <0.001 |
| Age at surgery | | | | | | |
| <60 years | 1 | | | 1 | | |
| 60–69 years | 1.52 | 1.05–2.23 | 0.025 | 1.52 | 1.05–2.19 | 0.027 |
| ≥ 70 years | 1.74 | 1.15–2.65 | 0.009 | 1.75 | 1.14–2.67 | 0.010 |
| Unkknown | 1.46 | 0.87–2.45 | 0.148 | | | |

12% for nm23-H1-negative patients. These findings are completely opposed to those reported by Mandai et al (1994), Viel et al (1995) and ourselves. We already described in a previous pilot study that nm23 expression, measured by means of immunohistochemistry, seemed to be associated with tumour aggressiveness and a trend towards lower survival (Schneider et al, 1996). This

 *© 2000 Cancer Research Campaign*

**Table 5** Analysis restricted to well or moderately differentiated tumours and stages I and II. Predictors of survival in ovarian cancer according to the multivariate nalysis

| Variable | Hazard ratio | 95% Confidence interval | *P*-value |
|---|---|---|---|
| nm23 expression | | | |
| 0–3 | 1 | | |
| 4–6 | 15.4 | 2.29–104.0 | 0.005 |
| Surgical stage | | | |
| I | 1 | | |
| II | 0.33 | 0.06–1.93 | 0.219 |
| Histology | | | |
| Serous-papillary | 1 | | |
| Mucinous | 0.49 | 0.08–3.04 | 0.446 |
| Endometrioid | 1.66 | 0.32–8.76 | 0.548 |
| Age at surgery | | | |
| <60 years | 1 | | |
| 60–69 years | 1.25 | 0.28–5.55 | 0.773 |
| ≥ 70 years | 0.96 | 0.16–5.72 | 0.965 |
| Unkknown | – | – | – |

tendency has been completely confirmed by the findings of the present series, the largest published to date on nm23 expression in ovarian cancer. Moreover, the statistical analysis of our study clearly shows that the association with a worse prognosis is stable over time, and mainly due to an effect on the subgroup of patients with a relatively better prognosis at the outset, i.e. those with well- or moderately differentiated tumours at earlier stages of the disease. Admittedly, our subgroup of early-stage cancers is relatively small (57 patients), if compared to the rest, but this only reflects the well-known fact that approximately 80% of ovarian cancer patients are diagnosed at advanced stages. Although the results obtained by us in this patient group have stood the test of multivariate analysis, nm23-overexpression being the only prognostic factor to retain statistical significance, they should be definitely confirmed in a larger series of patients with early-stage ovarian cancers. For them, a marker defining a high risk subpopulation would be of extreme value. In fact, according to most standard treatment protocols (Trimbos et al, 1991), there is no need to further treat most surgically staged patients with a confirmed early (I to IIA) stage, if their invasive ovarian cancers are well- to moderately differentiated. Our results, however, would suggest that precisely among this group of patients, a subgroup overexpressing nm23 and a much worse prognosis may be identified, who might probably benefit from some kind of adjuvant treatment.

For the nm23-H1 gene in human tumours, finally, both allelic deletion or point mutation seem not to affect prognosis. The only exception is perhaps childhood neuroblastoma, where overexpression of the mutant protein together with loss of heterozygosity, has been associated with a significantly worse prognosis (Leone et al, 1993). Although somatic allelic deletions of the nm23-H1 gene have been reported for a variety of human cancers, the association of this finding with a worse prognosis is far from evident in most cases (Leone et al, 1991b). The same can be said of somatic mutations of the gene, which have indeed been found in a variety of human tumours, but seem not to play a significant prognostic role, or give rise to contradictory reports. A particularly striking example in this respect is colon carcinoma, where some authors have found a significant correlation between genetic alterations and a metastatic phenotype (Wang et al, 1993), whereas others conclude that mutations play a negligible role in this tumour (Bafico et al, 1993). Fortunately, the results from the few studies on nm23-H1 that have been performed up to date in ovarian cancer are rather coincident in that mutations seem to play no significant role. So, allelic deletions in the long arm of chromosome 17 were studied by Leary et al (1995) and by Viel et al (1995). Both found an extremely high frequency of LOH (73–93%) in the tumours studied, without this having apparently any impact on prognosis or the metastatic behaviour of the tumours. They conclude that, from their results, the concept that nm23-H1 might be a suppressor gene cannot be supported. Although their results differ slightly from the just reported ones in that they found a lower rate of LOH at the nm23 locus, the conclusions of Mandai et al (1994) as to the lack of a suppressor role for nm23 are exactly the same. They searched for mutations in both nm23-H1 and nm23-H2, and found only one mutation among 35 invasive ovarian tumours, located in the nm23-H2 gene. After analysing 60 specimens by means of SSCP analysis and direct sequencing, we too have been able to identify only one point mutation, located in the untranslated region of exon 1. According to these data, somatic mutations in the nm23-H1 gene are also a rather uncommon feature in ovarian carcinoma.

All in all, our results, as well as those from the other available studies on nm23-H1 in human ovarian cancer point towards the fact that this gene does not function as a classic suppressor gene in this sort of tumour. If one should judge from the association between expression of the gene and tumour progession, it seems rather to act as an oncogene, or at least to be an adjuvant to, or a byproduct of, oncogenic activation. Since nm23-H2 has been shown in vitro to initiate transcription of c-*myc* (Postel et al, 1993), it is at least theoretically possible that nm23-H1 exerts a similar action on the same, or a related, oncogene in human ovarian cancer that could explain its repeated association with a more malignant phenotype. That it is indeed associated with the development of a more malignant phenotype in ovarian cancer is strongly suggested by our results, according to which the negative impact of nm23 expression was most evident in the subgroup of patients hosting theoretically less aggressive tumours, where it defined a subpopulation with significantly worse outcome, and revealed itself as the only independent predictor of survival.

### ACKNOWLEDGEMENTS

This work was supported by research grant P195/75 from Departamento de Educación, Universidades e Investigación del Gobierno Vasco, Spain. E. Jiménez and N. Martínez are recipients of predoctoral grants from Departamento de Educación, Universidades e Investigación del Gobierno Vasco, Spain.

### REFERENCES

Bafico A, Varesco L, De Benedetti L, Caligo MA, Grismondi V, Sciallero S, Aste H, Ferrara GB and Bevilacqua G (1993) Genomic PCR-SSCP anlysis of the metastasis associated nm23-H1 (NME1) gene: a study on colorectal cancer. *Anticancer Res* **13**: 2149–2154

Bevilacqua G, Sobel ME, Liotta LA and Steeg PS (1989) Association of low nm23 RNA levels in human primary infiltrating ductal breast carcinomas with lymph node involvement and other histopathological indicators of high metastatic potential. *Cancer Res* **49**: 5185–5190

Breslow EN and Day EN (1987) The Analysis of Cohort Studies. In: *Statistical Methods in Cancer Research, Vol II*. IARC Scientific Publications, Lyon

Caligo MA, Cipollini G, Fiore L, Calvo S, Basolo F, Collecchi P, Ciardello F, Pepe S, Petrini M and Bevilacqua G (1995) NM23 gene expression correlates with cell growth rate and S-phase. *Int J Cancer* **60**: 837–842

© 2000 Cancer Research Campaign

Chang CL, Zhu XX, Thoraval DH, Ungar D, Rawwas J, Hora N, Strahler JR, Hanasch SM and Radany E (1994) Nm23-H1 mutation in neuroblastoma. *Nature* **370**: 335–336
Florenes VA, Aamdal S, Myklebost O, Maelandsmo GM, Bruland OS and Fostad O (1992) Levels of nm23 messenger RNA in metastatic malignant melanomas: inverse correlation to disease progression. *Cancer Res* **52**: 6088–6091
Gilles AM, Presecan E, Vonica A and Lascu I (1991) Nucleoside diphosphate kinase from human erythrocytes. Structural characterization of the two polypeptide chains responsible for heterogeneity of the hexameric enzyme. *J Biol Chem* **266**: 8784–8789
Hailat N, Keim DR, Melhem RF, Zhu XX, Eckerskorn C, Brodeur GM, Reynolds CP, Seeger RC, Lottspeich F, Strahler JR and Hanash SM (1991) High levels of p19/nm23 in neuroblastoma are associated with advanced stage disease and with N-myc gene amplification. *J Clin Invest* **88**: 341–345
Hennessy C, Henry JA, May FEB, Westley BR, Angus B and Lennard TWJ (1991) Expression of the antimetastatic gene nm23 in human breast cancer: an association with good prognosis. *J Natl Cancer Inst* **83**: 281–285
Hess KR (1995) Graphical methods for assessing violations of the proportional hazards assumption in Cox regression. *Stats Med* **14**: 1707–1723
Howlett AR, Petersen OW, Steeg PS and Bissell MJ (1994) A novel function for the nm23 gene: overexpression in human breast carcinoma cells leads to the formation of basement membrane and growth arrest. *J Natl Cancer Inst* **86**: 1838–1844
Kapitanovic S, Spaventi R, Vujsic S, Petrovic Z, Kurjak A, Pavelic ZP, Glucksman JL, Stambrook PJ and Pavelic K (1995) nm23-H1 gene expression in ovarian tumors – a potential tumor marker. *Anticancer Res* **15**: 587–590
Leary JA, Kerr J, Chenevix-Trench G, Doris CP, Hurst T, Houghton CR and Friedlander ML (1995) Increased expression of the NME1 gene is associated with metastasis in epithelial ovarian cancer. *Int J Cancer* **64**: 189–195
Leone A, Flatow U, King CR, Sandeen MA, Margulles IM, Liotta LA and Steeg PS (1991*a*) Reduced tumor incidence, metastatic potential, and cytokine responsiveness of nm23-transfected melanoma cells. *Cell* **65**: 25–35
Leone A, McBride OW, Weston A, Wang MG, Anglard P, Cropp CS, Goepel JR, Lidereau R, Callahan R, Linehan WM, Rees RC, Harris CC, Liotta LA and Steeg PS (1991*b*) Somatic allelic deletion of nm23 in human cancer. *Cancer Res* **51**: 2490–2493
Leone A, Flatow U, Van Houtte K and Steeg PS (1993) Transfection of human nm23H1 into human MDA-MB-435 breast carcinoma cell line: effects on tumour metastatic potential, colonization and enzymatic activity. *Oncogene* **8**: 2325–2333
Lombardi D, Sacchi A, D'Angostino G and Tibursi G (1995) The association of the Nm23-H1 protein and beta-tubulin correlates with cell differentiation. *Exp Cell Res* **217**: 267–271
Mandai M, Konishi I, Koshiyama M, Mori T, Arao S, Tashiro H, Okamura H, Nomura H, Hiai H and Fukumoto M (1994) Expression of metastasis-related nm23-H1 and nm23-H2 genes in ovarian carcinomas: correlation with clinicopathology, EGFR, c-erbB-2, and c-erbB-3 genes, and sex steroid receptor expression. *Cancer Res* **54**: 1825–1830
Mandai M, Konishi I, Komatsu T, Mori T, Arao S, Nomura H, Kanda Y, Hiai H and Fukumoto M (1995) Mutation of the nm23 gene, loss of heterozygosity at the nm23 locus and K-ras mutation in ovarian carcinoma: correlation with tumor progression and nm23 expression. *Br J Cancer* **72**: 691–695
Martínez N, Jiménez E, Castresana JS and Schneider J (1997) Detection of p53 mutations in gynecologic tumors by means of a simple, nonradioactive minigel method of PCR-SSCP analysis. *Oncol Rep* **4**: 359–361
Nakamori S, Ishikawa O, Ohhigashi H, Kameyama M, Furukawa H, Sasaki Y, Inaji H, Higashiyama M, Imaoka S, Iwanaga T, Funai H, Wada A and Kimura M (1993) Expression of nucleoside diphosphate kinsase/nm23 gene product in human pancreatic cancer: an association with lymph node metastasis and tumor invasion. *Clin Exp Metastasis* **11**: 151–158
Nakayama H, Yasui W, Yokozaki H and Tahara E (1993) Reduced expression of nm23 is associated with metastasis of human gastric carcinomas. *Jpn J Cancer Res* **84**: 184–190
Nakayama T, Ohtsuru A, Nakao K, Shima M, Nakata K, Watanabe K, Ishii N, Kimura N and Nagataki S (1992) Expression in human hepatocellular carcinoma of nucleoside diophosphate kinase, a homologue of the nm23 gene produsct. *J Natl Cancer Inst* **84**: 1349–1354
Okabe-Kado J, Kasukabe T, Honma I, Hayashi M, Henzel WJ and Hozumi M Honma I, et al (1992) Identity of a differentiation inhibiting factor for mouse myeloid leukemia cells with nm23/nucleotide diphosphate kinase. *Biochem Biophys Res Commun* **182**: 987–994
Ozeki I, Takishima K and Mamiya G (1994) Immunohistochemical analysis of nm23/NDP kinase expression in human lung adenocarcinoma: association with tumor progression in Clara cell type. *Jpn J Cancer Res* **85**: 840–846
Postel EH, Berberich SJ, Flint J and Ferrone CA (1993) Human c-myc transcription factor PuF identified as nm23-H2 nucleoside diphosphate kinase, a candidate suppressor of tumour metastasis. *Science* **261**: 478–480
Royds JA, Stephenson TJ, Rees RC, Shothouse AJ and Silcocks PB (1993) nm23 protein expresson in ductal in situ and invasive human breast carcinoma. *J Natl Cancer Inst* **85**: 727–731
Royds JA, Cross SS, Silcocks PB, Scholefield JH, Rees RC and Stephenson TJ (1994) nm23 anti-metastatic gene product expression in colorectal carcinoma. *J Pathol* **172**: 261–266
Sastre-Garau X, Lacombe ML, Jouve M, Véron M and Magdalénat H (1992) Nucleoside diphosphate kinase/nm23 expression in breast cancer: lack of correlation with lymph-node metastasis. *Int J Cancer* **50**: 533–538
Scambia G, Ferrandina G, Marone M, Benedetti Panici P, Giannitelli C, Piantelli M, Leone A and Mancuso S (1996) Nm23 in ovarian cancer: correlation with clinical outcome and other clinico-pathological and biochemical prognostic parameters. *J Clin Oncol* **14**: 334–342
Schneider J, Rubio MP, Barbazán MJ, Rodríguez-Escudero FJ, Seizinger BR and Castresana JS (1994*a*) P-glycoprotein, HER-2/neu and mutant p53 expression in human gynecologic tumors. *J Natl Cancer Inst* **86**: 850–855
Schneider J, Rubio MP, Rodríguez-Escudero FJ, Seizinger BR and Castresana JS (1994*b*) Identification of p53 mutations by means of single-strand conformation polymorphism analysis in gynaecological tumors: comparison with the results of immunohistochemistry. *Eur J Cancer* **30A**: 504–508
Schneider J, Romero H, Ruiz R, Centeno MM and Rodríguez-Escudero FJ (1996) nm23 expression in advanced and borderline ovarian carcinoma. *Anticancer Res* **16**: 1197–1202
Stahl JA, Leone A, Rosengard AM, Porter L, King CR and Steeg PS (1991) Identification of a second human nm23 gene, nm23-H2. *Cancer Res* **51**: 445–449
Steeg PS, Bevilacqua G, Kopper L, Thorgeirsson UP, Talmadge JE, Liotta LA and Sobel ME (1988) Evidence for a novel gene associated with low tumor metastatic potential. *J Natl Cancer Inst* **80**: 200–204
Trimbos JB, Schueler JA, van der Burg M, Hermans J, van Lent M, Heintz AP and Fleuren GJ (1991) Watch and wait after careful surgical treatment and staging in well-differentiated early ovarian cancer. *Cancer* **67**: 597–602
Viel A, Dall'Agnese L, Canzonieri V, Sopracordevole F, Capozzi E, Carbone A, Visentin MC and Boiocchi M (1995) Suppressive role of the metastasis related nm23-H1 gene in human ovarian carcinomas: association with high messenger RNA expression with lack of lymph node metastasis. *Cancer Res* **55**: 2645–2650
Wang L, Patel U, Ghosh L, Chen HC and Banerjee S (1993) Mutation in the nm23 gene is associated with metastasis in colorectal cancer. *Cancer Res* **53**: 717–720

 *© 2000 Cancer Research Campaign*
